# The Role of Oxygen Vacancies in Phase Transition and the Optical Absorption Properties within Nanocrystalline ZrO_2_

**DOI:** 10.3390/nano14110967

**Published:** 2024-06-02

**Authors:** Jing Ouyang, Yonghui Peng, Wentao Zhou, Xianfeng Liang, Gang Wang, Qi Zhang, Bo Yuan

**Affiliations:** 1 Key Laboratory for Mineral Materials and Application of Hunan Province, Department of Inorganic Materials, School of Minerals Processing and Bioengineering, Central South University, Changsha 410083, China; 215611071@csu.edu.cn (Y.P.); 225611020@csu.edu.cn (W.Z.); gxliangxianfeng@126.com (X.L.); 2 Engineering Research Center of Ministry of Education for Carbon Emission Reduction in Metal Resource Exploitation and Utilization, Central South University, Changsha 410083, China; 3 State Key Laboratory of Advanced Refractories, Luoyang 471039, China; wangg@lirrc.com (G.W.); zhangqlirr@163.com (Q.Z.)

**Keywords:** oxygen vacancies, energy bands, zirconia nanoparticles, solvothermal preparation

## Abstract

Zirconia (ZrO_2_) nanoparticles were synthesized using a solvothermal method under varying synthesis conditions, namely acidic, neutral, and alkaline. X-ray diffraction (XRD) and field emission scanning electron microscopy (FESEM) were leveraged to investigate the phase evolution and topographical features in detail. The resulting crystal phase structures and grain sizes exhibited substantial variation based on these conditions. Notably, the acidic condition fostered a monoclinic phase in ZrO_2_, while the alkaline condition yielded a combination of tetragonal and monoclinic phases. In contrast, ZrO_2_ obtained under neutral conditions demonstrated a refinement in grain sizes, constrained within a 1 nm scale upon an 800 °C thermal treatment. This was accompanied by an important transformation from a monoclinic phase to tetragonal phase in the ZrO_2_. Furthermore, a rigorous examination of XPS data and a UV-visible spectrometer (UV-vis) analysis revealed the significant role of oxygen vacancies in phase stabilization. The notable emergence of new energy bands in ZrO_2_, in stark contrast to the intrinsic bands observed in a pure monoclinic sample, are attributed to these oxygen vacancies. This research offers valuable insights into the novel energy bands, phase stability, and optical absorption properties influenced by oxygen vacancies in ZrO_2_. Moreover, it proposes an innovative energy level model for zirconia, underpinning its applicability in diverse technological areas.

## 1. Introduction

Zirconia (ZrO_2_), with its extensive applications including but not limited to thermal barrier coatings, catalysts or carriers, fuel cells, functional ceramics, and oxygen ion conductors, is notable for its unique polymorphism [1,2,3,4,5]. The key feature of zirconia’s polymorphism involves the transition among three crystalline structures: monoclinic (m), tetragonal (t), and cubic (c). These transitions occur under distinct thermal or strain-related conditions and often accompany a considerable change in volume. Intensive research has been conducted into achieving the stabilization of the ZrO_2_ lattice through the method of doping with selected metal ions [6,7,8,9,10,11]. These efforts are intended to preserve its invaluable properties, particularly for applications that involve high-temperature conditions. Moreover, the lower phonon energy of ZrO_2_, relative to that of the silica matrix (as is about 1100 cm^−1^ for SiO_2_ and 470 cm^−1^ for ZrO_2_) [12], makes it an attractive host for luminescent ions with smaller energy gaps. Another remarkable application of ZrO_2_ emerges in the field of micro/mesoporous materials. The catalytic properties of ZrO_2_ originate from several characteristics. These include moderate acidity, oxidizing capability, thermal stability, and an extraordinarily large specific surface area up to 1200 m^2^∙g^−1^ [13]. These catalytic activities can be amplified through surface modifications using anions such as SO_4_^2+^, S_2_O_8_^2+^, or PO_4_^3+^, resulting in the formation of solid superacids (also known as SSAs) [14,15,16]. Significant advancements in material fabrication techniques have been witnessed. These have enabled the development of ZrO_2_ morphologies that can be tailored for specific applications. For example, t-ZrO_2_ nanowires, produced using templating methods, have been used as optical storage media for high-density digital versatile discs (HD-DVDs) [17]. Additionally, the method of atomic layer deposition has facilitated the crafting of ZrO_2_ nanotubes, allowing for tunable wall thickness [18].

Nanomaterial research has acctracted increased focus due to the unique characteristics of these materials, such as the effects of quantum confinement and tunneling, which are not typically observed in bulk materials. The utilization of nanocrystals as building blocks for device construction is an important practical application, and several devices have been produced successful [19,20,21]. As a result, the synthesize of nanocrystals with controlled properties, such as size, shape, structure, and size distribution, is critical for nanomaterials area. this study focuses on ZrO_2_ nanomaterials. Microwave-assisted synthesis methods have demonstrated that metastable ZrO_2_, even without any stabilizers, can be achieved when the product reaches the nanoscale [22]. Advanced investigations combining ab initio calculations and transmission electron microscopy (TEM) further showed that cubic ZrO_2_ (c-ZrO_2_) can remain stable. This is true even when the nanocrystal sizes are reduced to less than 2 nm [23]. Tetragonal ZrO_2_ (t-ZrO_2_) also exhibited stability for crystal sizes between 15 and 60 nm, supporting the findings of previous research by R.C. Gervie that pure t-ZrO_2_ can be produced with diameters of less than 30 nm when it has excess free energy up to 6.4 kcal∙mol^−1^ [24]. In relation to the polymorphism of ZrO_2_ and its multi-faceted applications, the impact of thermal conditions on its crystal structure transitions is of a imperative task. This task assumes particular relevance for high-temperature applications, as thermal conditions potentially trigger transitions across monoclinic, tetragonal, and cubic structures [25,26]. The temperature of calcination, a broadly utilized thermal treatment technique, will influence both phase formation and structural stability in ZrO_2_ samples. Consequently, a rigorous examination of ZrO_2_ samples calcined at varying temperatures can illuminate the intricate relationship of these varied structures. Such an understanding holds potential to enhance precision in phase control during the synthesis and processing of ZrO_2_. Furthermore, more specific theoretical predictions, or empirical findings, connecting calcination temperature and phase control would be beneficial to substantiate this point of view. Given the potential applications of these nanomaterials, this study aims to synthesize and characterize pure ZrO_2_ nanoparticles with different structures using a solvothermal route under specificly controlled conditions. The outcomes indicate the successful production of ZrO_2_ nanospheres with diameter of approximately 10 nm. The stabilization mechanism for t-ZrO_2_ will be deeply analyzed, laying a foundation for future efforts in the preparation and characterization of nanomaterials.

## 2. Materials and Methods

**Figure 1:**
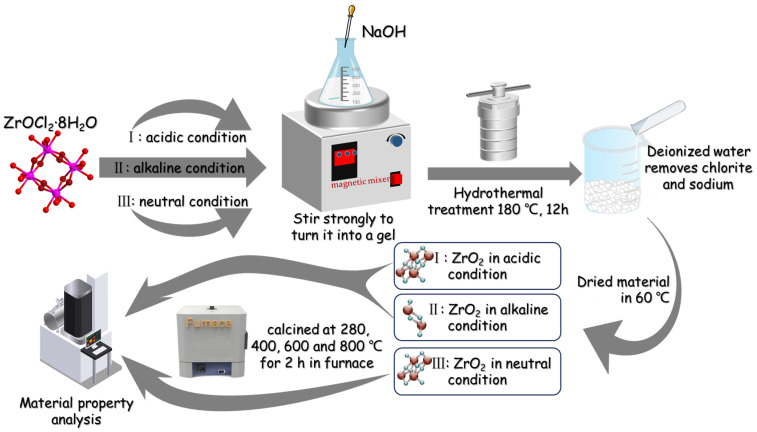
Schematic diagram of experimental process.

Zirconium chlorite (ZrOCl_2_∙8H_2_O) and NaOH were purchased from Aldrich, and were used directly as material sources without further purification. The raw sources underwent processing under three circumstances: (I) Under acidic conditions, with vigorous stirring, 0.01 mol of ZrOCl_2_∙8H_2_O was dispersed in 80 mL of pure ethanol. The mixture achieved a uniform sol state and was subsequently transferred into a Teflon-lined stainless steel autoclave. The reaction was conducted at 180 °C for 12 h before cooling in air. (II) Under alkaline conditions, the procedure was similar to that in step (I), but with an additional 0.025 mol of solid NaOH introduced into the mixture. (III) Under neutral conditions, 0.01 mol of ZrOCl_2_∙8H_2_O was dissolved in 100 mL of deionized water. Then, 0.01 mol∙L^−1^ of NaOH was slowly incorporated into the solvent with vigorous stirring until the mixture’s pH reached 7.5. The resulting white gel was centrifuged and washed with deionized water five times to remove any residual chlorite and sodium in the precursor. The precursor was then autoclaved following the methodology in process (I). The entirety of these procedures was carried out at room temperature. Bright white products were obtained from each process, which were then cleansed with both ethanol and deionized water, followed by air drying at 60 °C. For the thermal stability evaluation, the product obtained from process (III) was calcined in air at 280, 400, 600, and 800 °C for 2 h, respectively. For clarification, refer to Figure 1 for a detailed procedural illustration.

The as-prepared and sintered products were characterized using X-ray diffraction. This was performed with a RIGAKU D/max-2550VB^+^ 18 kW powder diffractometer and CuKα-radiation (λ = 1.541806 Å). Data were collected from 10° to 90° of 2*θ* with a step width of 0.02°. The phases were identified using the Search/Match capabilities of the JADE 5.0 program in conjunction with the ICDD (International Center for Diffraction Data) Powder Diffraction File (PDF) database. The morphology and lattice images of the nanocrystals were detected using FEI Sirion 200 field emission scanning electron microscopy (SEM). The SEM samples were prepared as follows: First, the as-prepared powder was dispersed in pure ethanol. Then, supersonic vibration was used to ensure an even distribution. This dispersion was then dropped onto brass stubs and dried at 60 °C. Next, the stubs were conductively coated with gold by sputtering for 10 s. This ensured an even and thin layer, which helped to minimize charging effects under SEM imaging. UV-vis absorption spectroscopy of the samples was conducted, and data were acquired using a Shimadzu UV-2450 UV-vis spectrophotometer. This analysis was an integral part of the characterization process, providing valuable information about the optical properties of the as-prepared and sintered samples. X-ray photoelectron spectroscopy (XPS) was employed to analyze the chemical circumstance and elemental composition of the obtained ZrO_2_ product. It was acquired on a Thermo Scientific K-Alpha, USA, with a hemispherical electron analyzer and an Al Ka X-ray radiation source. The pressure in the analysis chamber was <10^−9^ Torr. XPS binding energy (BE) was internally referenced to the C 1 s peak (BE = 284.6 eV). 

## 3. Results and Discussion

**Figure 2:**
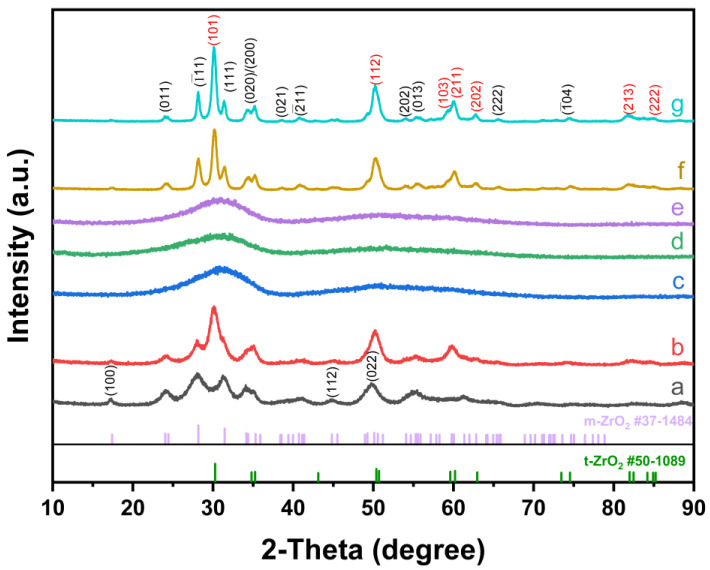
XRD patterns of sample yielded (**a**) under acidic condition, (**b**) under alkaline condition, (**c**) under neutral condition, and calcined at (**d**) 280 °C, (**e**) 400 °C, (**f**) 600 °C, and (**g**) 800 °C.

Figure 2 shows the XRD patterns of the as-prepared samples and those after sintering at different temperature. The primary XRD peaks of the sample obtained under acidic conditions (a) are concentrated at 2*θ* values of approximately 17.2°, 24.2°, 28.2°, 31.4°, and 34.4°. These peaks are indexed to the (100), (011), ($\bar{1}$11), (111), and (200) planes of monoclinic ZrO_2_ (m-ZrO_2_), respectively. As inferred from the patterns, the product synthesized in acidic conditions manifests predominantly pure m-ZrO_2_ nanocrystals (reference: Joint Committee of Powder Diffraction Files, JCPDS No.37-1484), with an average diameter of 3.2 nm as per Scherrer’s equation. This outlines an efficacious approach to synthesize m-ZrO_2_ nanocrystals. Conversely, the XRD spectra of the samples derived under alkaline conditions (b) exhibit two distinct sets of peaks corresponding to the monoclinic and tetragonal phases. The primary peaks for the tetragonal phase are detected at 2*θ* approximate values of 30.2°, 34.8°, and 50.3°; which can be indexed to the (101), (112), and (103) planes of tetragonal ZrO_2_ (t-ZrO_2_) according to JCPDS No. 50-1089. The XRD patterns of the product obtained under neutral solvothermal conditions can be indexed to t-ZrO_2_, with a crystalline diameter of approximately 1.1 nm, although the diffraction peaks remain somewhat indistinct. Post-sintering at 280 °C and 400 °C for 2 h, the nanocrystals maintain their tetragonal structure, with distinctive diffraction peaks gradually taking shape from the previously flat pattern. Nonetheless, the products sintered at 600 °C and 800 °C reveal both tetragonal and monoclinic structures of ZrO_2_. This indicates the emergence of m-ZrO_2_ at the expense of t-ZrO_2_ nanocrystals, suggesting that the synthesized nanocrystals retain their stability up to 400 °C.

**Figure 3:**
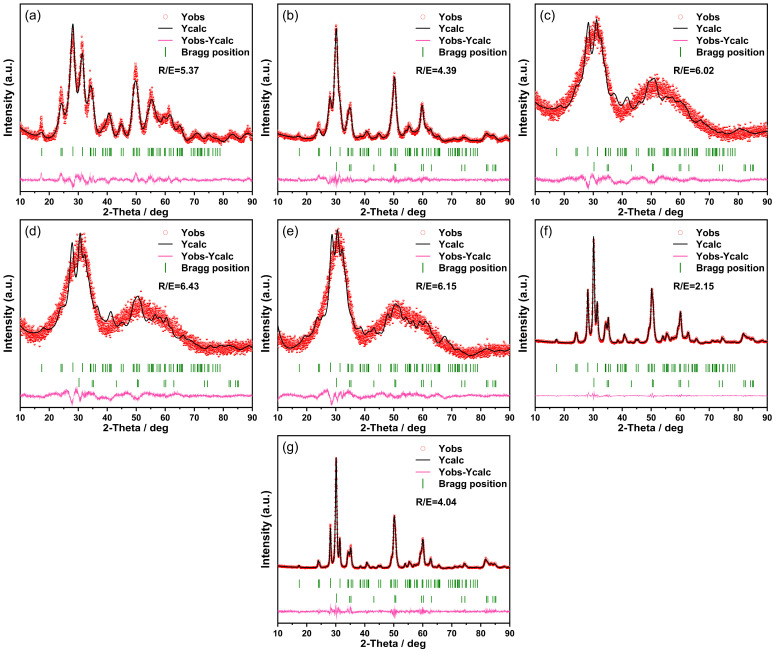
Rietveld analysis of XRD patterns: comparison of samples (**a**) under acidic condition, (**b**) under alkaline condition, (**c**) under neutral condition, and calcined at (**d**) 280 °C, (**e**) 400 °C, (**f**) 600 °C, and (**g**) 800 °C.

XRD patterns were quantitatively analyzed using Rietveld refinement to obtain more precise information on the crystal structures and phase compositions. Figure 3 presents the Rietveld analysis of the XRD spectra for samples prepared under diverse conditions and post-sintering treatment. The detailed outcomes from this refinement are disclosed in Table 1. In an acidic environment, the phase composition of synthesized pure ZrO_2_ appeared to be monoclinic. The space group of this monoclinic phase is mP21/c (14). However, under alkaline circumstances, the resulting pure ZrO_2_ exhibited both monoclinic and tetragonal phase compositions. The respective space groups are mP21/c (14) for the monoclinic phase and tP42/nmc2 (137) for the tetragonal phase, with their occurrence ratios being 52.8% and 47.2%. The ZrO_2_ samples synthesized under neutral conditions exhibited an amorphous structure with a minor presence of tetragonal phases below 400 °C, whilst predominantly showing a monoclinic phase composition. At room temperature, 280 °C, and 400 °C, the phase compositions did not present significant variations. The monoclinic phase proportion ranges from 83.3% to 83.6%, and the tetragonal phase accounts for 16.4% to 16.7%. Importantly, sintering temperatures beyond 400 °C triggered a phase transition in ZrO_2_. This temperature rise led to a detectable decrease in the monoclinic phase accompanied by a concomitant increase in the tetragonal phase. For instance, ZrO_2_ sintered at 600 °C demonstrated a phase constitution that was majorly tetragonal, accounting for 51.4%, coupled with a monoclinic component comprising 48.6%. Eventually, the tetragonal phase concentration soared to 61.5% in ZrO_2_ sintered at 800 °C, while the monoclinic phase proportion plummeted to 38.5%.

The causes of the noted disparities in the crystal phases among the samples obtained under various conditions are not yet fully elucidated. However, it can be conjectured from the empirical results that the environment surrounding the zirconium atoms may be responsible for the observed disparities. As demonstrated in previous studies, in ZrOCl_2_∙8H_2_O crystals, the tetrameric (Zr(OH)_2_·4H_2_O)_4_^8+^ group, rather than the zirconyl ions (ZrO^2+^), is the predominant zirconium state, with Cl^−^ acting as a coordinator to this species [27]. When (Zr(OH)_2_·4H_2_O)_4_^8+^ is dispersed in pure ethanol solvent and thermally treated at certain temperatures, the pronounced dehydration property of the solvent might cause the (Zr(OH)_2_·4H_2_O)_4_^8+^ group to gradually lose its coordinated H_2_O, consequently leading to dehydration and the release of H^+^ ions, resulting in the formation of ZrO_2_. This process can be represented by Equations (1) and (2). In the experimented acidic conditions, the insipiently produced H^+^ ions can be coordinated by Cl^−^ in the ethanol solvent and would not react with the ZrO_2_, which is evidenced by the markedly low pH (approximately 1.0) of the post-treatment solvent. Additionally, the presence of H^+^ ions would inevitably encourage the formation of H_3_O^+^, thereby impeding the formation of OH^−^ in the solvent.

(1) $${(\mathrm{Zr}(\mathrm{OH}{)}_{2}\cdot 4H_{2}O{)}_{4}}^{8+}\overset{\Delta }{\to }{(\mathrm{Zr}(\mathrm{OH}{)}_{2}{)}_{4}}^{8+}+16H_{2}O$$

(2) $${(\mathrm{Zr}(\mathrm{OH}{)}_{2}{)}_{4}}^{8+}\overset{\Delta }{\to }4\mathrm{Zr}O_{2}+8H^{+}$$

However, under alkaline conditions, the (Zr(OH)_2_·4H_2_O)_4_^8+^ group initially releases H^+^ ions from the coordinated water. This action consequently introduces some non-bridging –OH groups onto the zirconium atoms, leading to the formation of (Zr(OH)_2+x_·(4 − x)H_2_O)_4_^(8−4x)+^ groups and, subsequently, the (Zr_y_(OH)_2+x−2y_·zH_2_O)_n_ groups [27]. These processes can be demonstrated by Equations (3) and (4). During the solvothermal treatment process, ZrO_2_ results from the simple dehydration of the (Zr_y_(OH)_2+x−2y_·zH_2_O)_n_ groups, as shown in Equation (5). Under these circumstances, Cl^−^ is coordinated by Na^+^ through electrostatic forces upon their initial contact, leaving an excess of OH^−^ ions. These alkaline OH^−^ ions may coordinate with the resulting ZrO_2_ and, in selected instances, may infiltrate the ZrO_2_ lattice to form Zr atoms with eight-fold coordination, thereby forming tetragonal ZrO_2_. Meanwhile, Zr atoms with seven-fold coordination by oxygen atoms form monoclinic ZrO_2_, accounting for the observed presence of a mixture of t- and m- ZrO_2_ in samples synthesized under alkaline conditions.

(Zr(OH)_2_·4H_2_O)_4_^8+^ → (Zr(OH)_2+x_·(4 − x)H_2_O)_4_^(8−4x)+^ + 4xH^+^ (3)

(Zr(OH)_2+x_·(4 − x)H_2_O)_4_^(8−4x)+^ → (Zr_y_(OH)_2+x−2y_·zH_2_O)_n_↓ + mH_2_O (4)

(5) $$({\mathrm{Zr}}_{y}(\mathrm{OH}{)}_{2+x-2y}\cdot {\mathrm{zH}}_{2}O{)}_{n}\overset{\Delta }{\to }\mathrm{nyZr}O_{2}+1H_{2}O$$

In the sediments repeatedly washed with deionized water, neither the Cl^−^ and Na^+^ ions, nor the excessive H^+^ and OH^−^ ions will present in the precursor. Therefore, the solvothermally treated (Zr_y_(OH)_2+x−2y_·zH_2_O)_n_ groups will only undergo dehydration to form ZrO_2_, as reflected in Equation (5). Compared to the samples prepared under acidic and alkaline conditions, the sample synthesized under neutral conditions exhibits an ultra-fine crystal size (approximately 1.1 nm), indicating that the absence of an ionic environment impedes the growth of nanocrystals. This may also be due to the presence of excess ions, as the high solubility of the sub-critical (with the critical temperature for ethanol being 249.4 °C [28]) organic solvent, ions in the solvent may act as ‘transporters’ [29], which will facilitate the growth of solid substances in solvothermal experiments, as proposed by S. Tsunekawa et al. Consequently, the loaf-like diffraction peaks suggest the presence of tetragonal phase ZrO_2_ [23].

**Figure 4:**
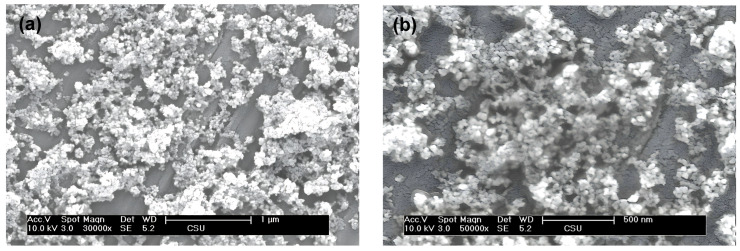
(**a**,**b**) SEM images of pure ZrO_2_ nanoparticles under neutral condition.

In the absence of surplus ions adsorbed on, or coordinating with, the freshly prepared pure ZrO_2_, the products are expected to attain a higher purity level compared to the other two variants. Consequently, morphological characterization was conducted on the sample synthesized under neutral conditions, as illustrated in SEM images in Figure 4. Insights from Figure 4 reveal that the minute particles feature a relatively narrow size distribution. However, several agglomerates with an average diameter of approximately 400 nm are also discernible. This phenomenon can be ascribed to the vast surface area and surface free energy of the nanoparticles. The exceedingly large values of these two parameters invariably result in the agglomeration of nano-scale particles to minimize their surface energy.

**Figure 5:**
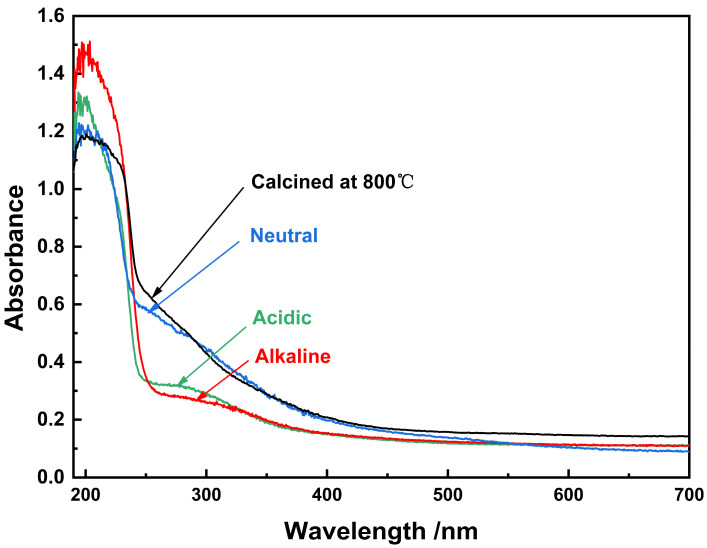
UV-vis absorption spectra of pure ZrO_2_ samples under different conditions.

The UV-visible absorption spectra of the samples derived from various conditions are displayed in Figure 5. Each of the specimens demonstrates absorption bands centered near the wavelength of 200 nm within the UV light region, indicative of the intrinsic energy bands of ZrO_2_. Less pronounced absorption is further noted in the range between 250 nm and 300 nm. An integration of the UV-visible spectroscopic observations with an X-ray diffraction (XRD) phase analysis reveals that the ZrO_2_ sample, synthesized in acidic conditions, presents its dominant absorption band at a decreased wavelength, reflecting the maximum band gap energy. This can be ascribed to the ZrO_2_ being, in its entirety, a pure monoclinic phase. In contraste, the shift in the absorption edge towards extended wavelengths for the samples synthesized under alkaline and neutral conditions implies a contraction in the band gap energy. This phenomenon might be due to a marginal phase shift from monoclinic ZrO_2_ (m-ZrO_2_) to tetragonal ZrO_2_ (t-ZrO_2_), yielding a slight number of oxygen vacancies. The introduction of these vacancies generates new defect energy levels in the band gap, forming so-called band gap states, which subsequently diminish the material’s direct band gap, causing a red shift on the absorption edge [30,31]. However, following the calcination of the ZrO_2_ sample at 800 °C for 2 h, the wavelength of the main absorption band becomes significantly shortened, rendering the band’s energy position at the zenith, thereby inducing a blue shift at the absorption edge. This occurrence can be ascribed to the elevated quantity of monoclinic ZrO_2_ transitioning to the more heat-stable tetragonal variant following the high-temperature calcination process, in which the lattice’s zirconium ions are displaced, forming abundant oxygen vacancies. As such, these vacancies result in the introduction of copious new defect energy levels in the lattice, which, due to overlap, lead to an increase in direct bandgap energy [32]. Additionally, a high prevalence of oxygen vacancies could potentially trigger a reduction in grain size, causing a further amplification of the direct band gap energy (due to the quantum size effect) [33]. The culmination of these factors consequently results in the blue shift of the absorption edge.

The band structure of zirconia is complex, and a variety of methodologies have been utilized for its elucidation. These techniques include vacuum ultraviolet spectroscopy (VUV), electron energy-loss spectroscopy (EELS), valence band X-ray photoelectron spectroscopy (VBXPS), and ab initio calculations. These methods have generated a multitude of results and interpretations. In their previous work, Fan and colleagues [34] adapted optical absorption spectra of m-ZrO_2_ and m + t-ZrO_2_ films fabricated via sputter deposition, identified two direct band transitions at energies of 5.79 eV and 5.20 eV. Contrastingly, an indirect interband transition was observed at approximately 4.7 eV for m-ZrO_2_ films [35]. Considering the electronic structure, the hybridization or mixing of Zr 4d (x^2^ − y^2^ and z^2^) and Zr 4d (xy, xz, and zx) orbitals could result in differently structured conduction bands for the m-, t-, and c-ZrO_2_ phases. The resulted direct band gaps were measured to be 5.83 eV, 5.78 eV, and 6.1 eV for the first band, while 7.09 eV, 6.62 eV, and 7.08 eV were observed for the second bands, respectively. However, theoretical calculations indicate lower energy gap values of 4.46 eV, 4.28 eV, and 4.93 eV for these phases, respectively. Notably, for all phases, optical conductivity measurements revealed an interband transition at approximately 8 eV, which has been attributed to the O 2p to Zr 4d transition.

**Figure 6:**
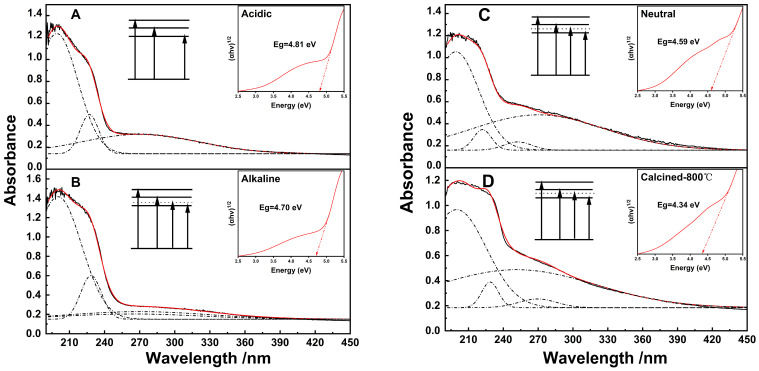
UV-vis spectra and their fitting lines for samples under different conditions, and their corresponding Tauc plots ((**A**) acidic, (**B**) alkaline, and (**C**) neutral conditions and (**D**) that calcined sample).

As demonstrated in the ultraviolet-visible absorption spectra, the absorption bands serve as markers for the absorption of photons of corresponding energies by the sample lattice, thereby facilitating a transition to an excited state for the electrons. An insightful representation is the spectra under different analyses conditions, as depicted by the Gaussian–Lorentz function fitting performed on the UV-vis spectra and their corresponding Tauc plot (Figure 6). Comprehensive details of the fitting line positions and indirect band gap energies can be found in Table 2. A critical scrutiny of the Tauc plot manifests notable variations in the indirect band gap of ZrO_2_, a finding attributable to the different preparation methods and conditions. To elaborate, the indirect band gaps of ZrO_2_ under acidic, alkaline, and neutral conditions are observed to be 4.81 eV, 4.70 eV, and 4.59 eV, respectively. After roasting, the gap was recorded as 4.37 eV. The observed red shift in the indirect band gap is proposed to be an outcome of structural dynamic transforms in ZrO_2_, presumably induced by differences in the preparation environment. The ensuing variation, specifically changes in oxygen vacancies, have been found to introduce additional states by acting as electron donors in ZrO_2_ samples [36]. These intermediate states in the band gap encouraged carrier transitions at lower energies, consequently reducing the indirect band gap. According to these findings, an electron excitation model was formulated for each sample based on the fitting lines. In the established model, each line is indicative of a state of the electron, a lower position representing a lower-energy state, and *vice versa*. The basic and elevated positions of the model represent the ground and excited states, respectively, with arrows depicting the possible electron transitions. Figure 6 and Table 2 reveal the presence of three immovable fitting lines in all the spectra, perfectly aligning with the fitting lines of the spectra obtained under the acidic condition. This observation suggests the existence of three intrinsic states in the electronic structure of the fabricated pure ZrO_2_ samples under varying conditions. Notably, the intrinsic states and the ground state are demarcated by solid lines in the electron excitation model. Interestingly, the UV-vis spectra of the samples acquired under non-acidic conditions showcased the appearance of a new fitting line, indicating the existence of a new states. These excited states are earmarked by dashed lines in the model (Figure 6), which was attributed to the states of oxygen vacancies in the fabricated ZrO_2_. Furthermore, the newly identified states are located between 5.44 eV and 4.59 eV, while the intrinsic states demonstrate energies of 6.22 eV, 5.44 eV, and 4.59 eV, respectively. Contrasted with the transitions presented by Fan and associates [34], the examined samples depict a blue shift at the band edge of the direct transition, coupled with a red shift in the indirect bandwidth gap. This observation is in agreement with the analysis shown in Figure 6.

**Figure 7:**
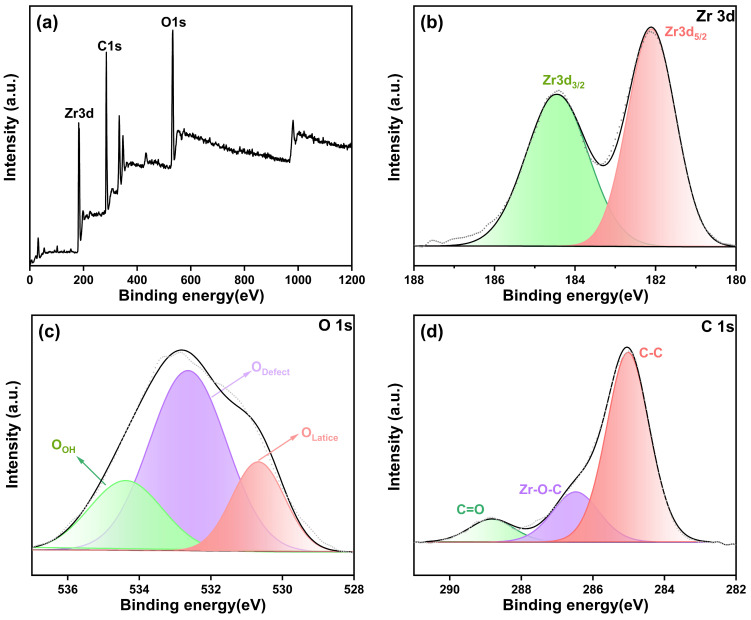
XPS spectra of calcined sample of (**a**) all elements, (**b**) Zr3d_3/2_ and Zr3d_5/2_, (**c**) O1s, and (**d**) C1s.

**Figure 8:**
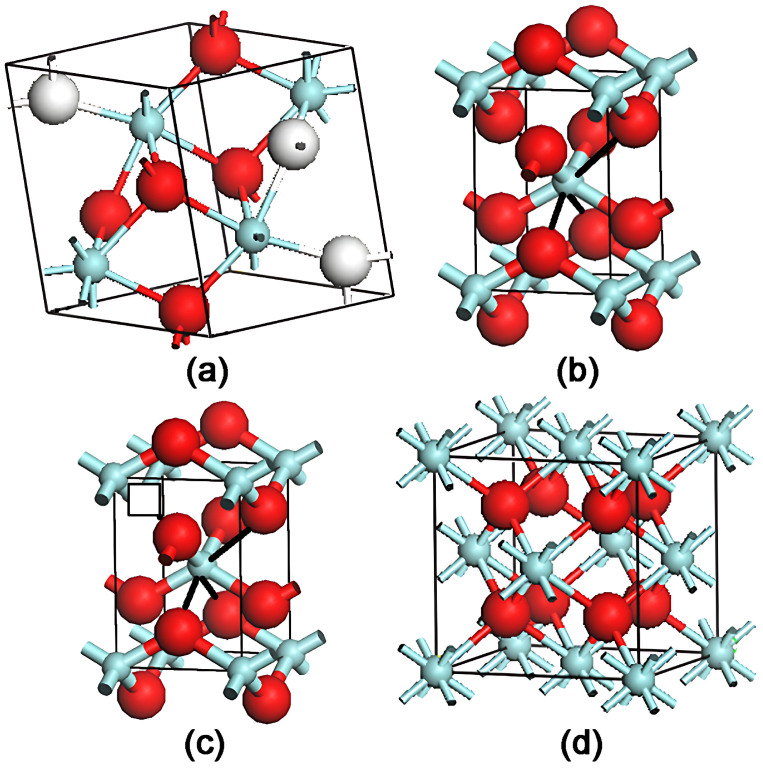
A depiction of crystal structures for (**a**) m-ZrO_2_, (**b**) t-ZrO_2_, (**c**) pseudo t-ZrO_2_, and (**d**) c-ZrO_2_. The red balls represent the O atoms, and the cyan balls represent the Zr atoms. The presence of an oxygen vacancy in the lattice is illustrated by the square in (**c**). The heavy black bonds in (**b**,**c**) arbitrarily connect O and Zr atoms for the purpose of indicating the nature of Zr atoms’ eight-fold coordination. In the monoclinic structure, the three-fold coordinated oxygen ions are appropriately labeled by white balls.

X-ray photoelectron spectroscopy (XPS) was employed to analyze the compositional elements and chemical states that constitute the ZrO_2_ nanostructure in the sample, which was subjected to calcination at 800 °C under neutral conditions. The fitting of the XPS curve for the ZrO_2_ surface is represented in Figure 7, and the relative concentration of surface oxygen species deduced from the XPS data is tabulated in Table 3. The broad spectrum depicted in Figure 7a exhibits prominent peaks at 184.95, 523.52, and 285.09 eV are correspond to Zr 3d, O 1s, and C 1s electrons, respectively. Notably, the high-resolution spectra of the Zr 3d peak (Figure 8b) reveal two distinct peaks at 182.10 and 184.44 eV, which can be attributed to the 3d_5/2_ and 3d_3/2_ energy states of Zr (IV) species, which are associated with the Zr-O bond [37,38]. The Zr3d_5/2_ binding energy is relevant to the monoclinic and tetragonal phases of ZrO_2_ [39]. Moreover, the O 1s spectra shown in Figure 7c show a broad range from 528 to 537 eV. Upon deconvolution, it further resolves into three distinct peaks at 530.76 eV, 533.03 eV, and 535.02 eV. As inferred from Figure 7 and Table 3, the binding energy peak at 530.76 eV is assigned to lattice oxygen species (O_Lattice_) on the surface. The moderate and higher energy peaks at 533.03 eV and 535.03 eV are attributed to chemically adsorbed oxygen species on the surface, denoted as O_Defect_ and O_OH_, respectively [40,41]. The proportions for O_Defect_ and O_OH_ were found to be 58.48% and 9.94%, respectively, indicating a significant increase in the quantity of oxygen vacancies present on the surface of the sample after calcination. Additionally, the high-resolution C 1s spectrum (Figure 8d) unveils three distinct peaks situated at 284.99 eV, 286.47 eV, and 288.80 eV, which are attributed to the C-C, Zr-O-C, and C=O functional groups, respectively [42].

Oxygen vacancies are integral to the stabilization and physical properties of zirconia. In m-ZrO_2_, zirconium atoms exist in a seven-fold coordination scheme surrounded by two types of non-equivalent oxygen sites, as illustrated in Figure 8. Two distinct arrangements are observed for the oxygen atoms: a tetragonal arrangement for half of them and a trigonal arrangement for the others. Unlike m-ZrO_2_, c-ZrO_2_ adopts a fluorite structure with Zr atoms forming a *bcc* lattice. Figure 8d shows oxygen atoms adopting a tetrahedral coordination towards Zr atoms, which are eight-fold coordinated. The nature of t-ZrO_2_ is characterized by a slight distortion from c-ZrO_2_, which involves an expansion of the cubic cell in the *c*-direction and a minor displacement of O atoms along the same axis, while the Zr still has an eight-fold coordination. Under ambient conditions, m-ZrO_2_ is the only stable phase. The introduction of stabilizers, such as Y^3+^, Ca^2+^, and others at specific dosages into the ZrO_2_ lattice, can lead to the induction of a substantial number of oxygen vacancies [43,44]. As depicted in Figure 8c, these vacancies, whether uniformly or unevenly dispersed in the oxygen sub-lattice, coordinate with zirconium atoms to form a ‘pseudo’ eight-fold coordination, promoting the stability of the c- or t-ZrO_2_ lattice at room temperature. It is relatively common for oxygen vacancies to persist in ZrO_2_ nanoparticles, given the inherent high density of defects in nanoscale materials, as well as in bulk ZrO_2_ materials. These vacancies introduce a new energy level into the gap of intrinsic bands, denoted by the dotted lines in Figure 6. Consequently, characteristic UV-vis absorption spectra from differently prepared samples were detected, with three intrinsic bands and a movable band between direct and indirect band bottoms [45]. The energy band models in Figure 6 and the phase compositions deduced from the XRD results suggest that a pure monoclinic sample has no (or very less) oxygen vacancies. Conversely, a significant number of vacancies are likely present in other samples with different structures, as is apparent from the emergence of UV light absorption signals linked to vacancies. As previously noted, these vacancies are instrumental in stabilizing t-phase zirconia in Figure 8c and bring about new bands in intrinsic bands [46].

## 4. Conclusions

The insights garnered from this study highlight the effect of oxygen vacancies on the phase and optical absorption properties of ZrO_2_ nanoparticles, which were synthesized through a solvothermal route under exclusive conditions, verified using tools such as X-ray diffraction, X-ray photoelectron spectroscopy, and field emission scanning electron microscopy. Acidic and alkaline conditions yielded larger grain sizes, with neutral conditions resulting in ultrafine grain sizes, evidencing the restrictive impact of an ionic environment on nanocrystal development.

The inquiry predominantly centered on the analysis of optical absorption spectra by leveraging a UV-vis spectrometer, revealing a distinct phase reliance with the specific preparation conditions. Oxygen vacancies, identified within the nanoparticles, play a decisive role in phase stabilization and optical absorption occurrence. This study presents a theoretical model delineating the energy levels in zirconia nanoparticles, offering valuable knowledge concerning the synthesis and characteristics of nano-crystalline ZrO_2_. Oxygen vacancies cause alterations in the coordination numbers of zirconium atoms and oxygen atoms within distinct ZrO_2_ crystal structures. The monoclinic phase represents the stable structure of zirconia under ambient conditions, while hexagonal and cubic ZrO_2_ phases emerge upon the induction of oxygen vacancies as stabilizers. These vacancies introduce new energy levels into zirconia, affecting the absorption features of the spectra. These research findings offer significant potential for a multiplicity of applications, including thermal barrier coatings, catalysts, fuel cells, and functional ceramics.

## Figures and Tables

**Table 1:** Rietveld refinement results for the samples obtained at different temperature.

Sample	Phase	Space Group	Phase Fraction/%	Lattice Parameters/nm			Residual	
				a	b	c	Rwp/%	R/E
ZrO_2_ in acidic condition	Monoclinic ZrO_2_	mP21/c (14)	100	5.2281	5.2566	5.3646	10.56%	5.37
ZrO_2_ in alkaline condition	Monoclinic ZrO_2_	mP21/c (14)	52.8	5.1752	5.2158	5.3365	8.83%	4.39
	Tetragonal ZrO_2_	tP42/nmc2 (137)	47.2	3.6297	3.6297	5.1171		
ZrO_2_ in neutral condition	Monoclinic ZrO_2_	mP21/c (14)	83.3	4.9119	5.2679	5.4161	11.47	6.02
	Tetragonal ZrO_2_	tP42/nmc2 (137)	16.7	3.4629	3.4629	5.3115		
ZrO_2_ (neutral) calcined at 280 °C	Monoclinic ZrO_2_	mP21/c (14)	83.3	4.9593	5.1612	5.4228	13.13	6.43
	Tetragonal ZrO_2_	tP42/nmc2 (137)	16.7	3.4618	3.4618	5.2946		
ZrO_2_ (neutral) calcined at 400 °C	Monoclinic ZrO_2_	mP21/c (14)	83.6	4.7394	5.3893	5.477	12.72	6.15
	Tetragonal ZrO_2_	tP42/nmc2 (137)	16.4	3.5745	3.5745	5.2084		
ZrO_2_ (neutral) calcined at 600 °C	Monoclinic ZrO_2_	mP21/c (14)	48.6	5.1471	5.2005	5.314	7.99	2.15
	Tetragonal ZrO_2_	tP42/nmc2 (137)	51.4	3.596	3.596	5.1825		
ZrO_2_ (neutral) calcined at 800 °C	Monoclinic ZrO_2_	mP21/c (14)	38.5	5.1474	5.2013	5.315	8.51	4.04
	Tetragonal ZrO_2_	tP42/nmc2 (137)	61.5	3.6016	3.6016	5.1835		

**Table 2:** The peak positions of the fitting lines in Figure 6.

Labels in Figure 6	A	B	C	D
Indirect band gap	4.81 eV	4.70 eV	4.59 eV	4.34 eV
Peak positions (nm) and energy state	199.1948 (6.23 eV)	200.2167 (6.20 eV)	199.1706 (6.22 eV)	199.8592 (6.21 eV)
	227.0601 (5.46 eV)	228.4576 (5.43 eV)	222.0763 (5.58 eV)	228.6299 (5.43 eV)
	-	264.767 (4.68 eV)	252.5948 (4.91 eV)	251.3968 (4.93 eV)
	270.3473 (4.59 eV)	269.7686 (4.60 eV)	270.0315 (4.59 eV)	269.5722 (4.60 eV)

**Table 3:** XPS fitting results of calcined sample.

Deconvolution	Zr3d_5/2_	Zr3d_3/2_	O_Lattice_	O_Defect_	O_OH_	C-C	Zr-O-C	C=O
Binding energy/eV	182.10	184.44	530.76	533.03	535.02	284.99	286.47	288.80
Area/%	52.36	47.64	31.58	58.48	9.94	44.28	12.75	5.60

## Data Availability

The data are contained within the article.
